# Antibiofilm Activity of a Thermoformable Material Loaded With Essential Oil Solution

**DOI:** 10.1002/cre2.70444

**Published:** 2026-08-30

**Authors:** Alexandra Stähli, Markus Laky, Anton Sculean, Tino Töpper, Sigrun Eick

**Affiliations:** ^1^ Department of Periodontology, School of Dental Medicine University of Bern Bern Switzerland; ^2^ Division of Conservative Dentistry and Periodontology, University Clinic of Dentistry Medical University of Vienna Vienna Austria; ^3^ Bottmedical AG, Tech Park Basel Switzerland

**Keywords:** biofilm, clear aligner appliances, clear dental braces, minimum inhibitory concentration, oral bacteria

## Abstract

**Background:**

Interest in antimicrobial surfaces for dental devices, such as aligners and brackets, is growing. These surfaces are designed to retard biofilm formation. One approach is to load essential oils (EOs) into the material. The focus of this study was the antibacterial, anti‐biofilm, and anti‐inflammatory activities of an EO solution.

**Methods:**

The EO solution contained cinnamaldehyde, methyl salicylate, eucalyptol, limonene, and trans‐anethole dissolved in distilled water (dH_2_O) with polyglycerol‐4‐laurate/sabacate (basic solution). The positive control was 0.2% chlorhexidine digluconate (CHX). The antibacterial activity of the EO and the basic solution was tested against selected oral bacteria. Multispecies biofilms were formed on specimens of a cellulose‐based (test) material loaded with the EO solution or the basic solution, and on a polyurethane‐based (reference) material loaded with CHX. The level of IL‐8 released from gingival fibroblasts (GF) exposed to the solutions was measured.

**Results:**

The EO solution clearly inhibited bacterial growth. However, the basic solution also acted as a growth inhibitor at higher concentrations. The EO solution reduced the metabolic activity of the formed biofilm. In addition, the basic solution decreased biofilm mass and colony‐forming unit counts. The basic solution was not inferior to CHX on the reference material. However, only the basic solution, not the EO solution, suppressed the release of IL‐8 from GF when stimulated with bacteria.

**Conclusion:**

Applying essential oils (EOs) to surfaces may be an effective way to inhibit biofilm formation. The basic solution, which is polyglyceryl‐laurate‐based, exhibits anti‐biofilm and anti‐inflammatory properties. Further in vitro research should aim to gain a deeper understanding of the solubilizer's mechanisms and optimize the composition of the added essential oils before testing in clinical trials.

## 1 Introduction

In dentistry, removable splints are used to stabilize autotransplanted teeth (Isa‐Kara et al. 2011) or traumatized teeth (X. T. Wang et al. 2024). They are also widely used as aligners in orthodontic therapy and can produce acceptable results in cases of mild to moderate malocclusion (Robertson et al. 2020). Biofilm‐associated oral diseases such as caries and periodontal disease are extremely common worldwide (Kassebaum et al. 2014; Wen et al. 2022). These oral diseases result in serious health and economic consequences and significantly impact quality of life (Peres et al. 2019). An increase in the plaque index, along with a higher number of white spot lesions, has been reported in adolescents undergoing aligner treatment (Wang et al. 2024). Wearing of brackets or aligners modifies the oral microbiome. As was recently demonstrated, species richness, as measured by the Chao1 index, decreases in individuals wearing aligners (C. Wang et al. 2024). Levels of the inflammatory cytokines interleukin (IL)‐6 and IL‐8 increased in gingival crevicular fluid in patients undergoing aligner therapy 2‐3 weeks after beginning the procedure (Altındal et al. 2025). It can be assumed that the increase in inflammatory cytokines correlates with the biofilm levels. Consequently, there is interest in antimicrobial surfaces for dental devices, such as aligners or brackets. To reduce this risk, especially for clear aligners, it would be beneficial if the material had an antibacterial effect.

A few attempts have been made to incorporate antibacterial agents into the aligner material. Aligner material with high fluoride concentrations (Yan et al. 2023) or with incorporated chlorhexidine and zinc oxide (Cao et al. 2023) inhibited in vitro biofilm formation of *Streptococcus mutans*. However, in both approaches, a negative long‐term effect of the compounds cannot be excluded. For example, high fluoride concentrations are suggested to increase the risk of dental fluorosis (Hung et al. 2023; Wong et al. 2024), and chlorhexidine may cause extrinsic tooth staining (Poppolo Deus and Ouanounou 2022).

Another approach is to load natural compounds, particularly essential oils, to the material. Essential oils (EOs) are secondary plant metabolites, that are mixtures of volatile, lipophilic, odoriferous substances, and they are well known for their antioxidant, anti‐inflammatory and antimicrobial activities (de Sousa et al. 2023). A thermoformable cellulose‐based polymer was loaded with cinnamonaldehyde. The material was then exposed to cultures of *Streptococcus mitis*, *S. mutans* or *Staphylococcus epidermidis*. The growth of *S. epidermidis* was clearly inhibited while the effect on the streptococcal species was less pronounced (Worreth et al. 2022). Later, based on the selective screening of 20 EOs, additional EOs were added to the loading solution for the thermoformable cellulose‐based polymer (Astasov‐Frauenhoffer et al. 2023). Except for cinnamaldehyde, eucalyptol, limonene, and trans‐anethole added by methyl‐salicylate were solubilized as a 5% solution in polyglycerol‐4‐laurate/sebacate solubilizer in water (Astasov‐Frauenhoffer et al. 2023). There was again a growth delay for *S. mutans* and *S. mitis*, when the cellulose‐based material was loaded with the EO solution; however, the effect was not visible on a comparative polyurethan material that was unable to retain the EO solution (Astasov‐Frauenhoffer et al. 2023). Moreover, it was demonstrated that loading the 750 µm thick cellulose‐based material with EO solution did not negatively impact its mechanical properties. Furthermore, these properties were similar to those of a widely used polyurethane‐based reference material (Astasov‐Frauenhoffer et al. 2023).

Promising results were obtained with the thermoformable material loaded with EO solution, but only with single strains of streptococci. Further exact antimicrobial data was lacking. Thus, this study aimed to determine the minimum inhibitory concentrations of the EO solution against oral bacteria and test its ability to inhibit biofilm formation. To study biofilm formation, a defined multi‐species biofilm containing species associated with both caries and gingivitis was used. To determine the potential effect of the solubilizing solution (without EOs), it was also tested in the experiments. To exclude an inflammatory response to the loading solution, the level of IL‐8 released by gingival fibroblasts was quantified after exposing the fibroblasts to the loading solutions.

## 2 Materials and Methods

### 2.1 Test Solutions and Specimens

The test solution contained cinnamaldehyde, methyl salicylate, eucalyptol, limonene, and trans‐anethole in distilled water (dH_2_O) supplemented with polyglycerol‐4‐laurate/sebacate as a solubilizer (Astasov‐Frauenhoffer et al. 2023). Since the role of the essential oils and other additives needed to be specified, the solubilizing (basic) solution (dH_2_O with polyglycerol‐4‐laurate/sebacate) was also included. Additionally, a 0.2% chlorhexidine digluconate (CHX) solution (pharmacy of the Insel University Hospital Bern) served as a positive control.

In biofilm assays, 5‐mm‐diameter disks were used. The disks were made from a thermoformable, cellulose‐based polymer material (test material, Bottmedical AG, Basel, Switzerland) and a reference material. For the reference material, the widely used, polyurethane‐based Zendura FLX material (Bay Materials LLC, Fremont, CA, USA) was selected.

The disc specimens were exposed to different solutions. The test material was coated with a solution containing essential oils and with the basic solution. The reference material was exposed to a 0.2% CHX solution. dH_2_O was used as a control for both types of materials.

The day before the experiment, the test and reference specimens were bathed in a solution while shaking for 6 h. After briefly rinsing them in dH_2_O, the specimens were stored overnight at 4°C. The procedure resulted in five different groups:

a. Test material with test solution
b. Test material with basic solution
c. Test material control
d. Reference material with 0.2% CHX
e. Reference material control.

### 2.2 Microorganisms

The experiments included nine bacterial species: *Actinomyces naeslundii* ATCC 12104 and *Streptococcus gordonii* ATCC 10558 as early colonizers; *Streptococcus mutans* ATCC 25175 and *Streptococcus sobrinus* ATCC 33478 as caries‐associated species; and *Fusobacterium nucleatum* ATCC 25586, *Parvimonas micra* ATCC 33270, *Campylobacter rectus* ATCC 33238, *Prevotella intermedia* ATCC 25611, and *Tannerella forsythia* ATCC 43037 as bacteria present in gingival inflammation.

The bacterial strains were precultured on tryptone‐soya agar supplemented with sheep blood (TSAB, Thermo Fisher Scientific, Allschwil, Switzerland). For *T. forsythia*, the medium was supplemented with 10 mg/l N‐acetyl muramic acid (NAM, Merck KGaA, Darmstadt, Germany).

The cultures were incubated with 10% CO_2_ for *Streptococcus* sp. and *A. naeslundii*, or anaerobically. Then, the strains were suspended in 0.9% NaCl according to the McFarland standard 4.

### 2.3 Minimum Inhibitory Concentration (MIC) Determination

A twofold dilution series was made from the test solutions (test, basic, and 0.2% CHX) in dH_2_O. One hundred microliters were pipetted into each well of a 96‐well plate. The bacterial suspension was mixed with the cultivation media (doubled‐concentrated Wilkins‐Chalgren broth (Thermo Fisher Scientific) with 10 mg/ml β‐NAD (Merck)) at a ratio of 1: 50. Then, 100 µl were added to the wells containing the test substances. Thus, the final concentrations in the mixtures were 50%–0.05% of the original solutions.

The 96‐well‐plates were incubated for either 18 h with 10% CO_2_ (*Streptococcus* sp., *A. naeslundii*) or 24 h anaerobically. Thereafter, the MIC was measured using a microplate reader at 600 nm. The MIC was the lowest concentration at which no visible bacterial growth occurred. The experiments were performed as independent replicates.

### 2.4 Biofilm Formation

The prepared specimens were placed in 24‐well plates and coated with 100 µl of a 0.67% mucin solution (Merck KgaA) for 30 min. Meanwhile, a suspension mixture of bacteria was prepared by mixing one part *S. gordonii* with two parts of *A. naeslundii*, *S. mutans*, and four parts of the other species. The mixture was added to nutrient broth (Wilkins‐Chalgren‐broth with 10 mg/l β‐NAD and 10 mg/l NAM) in a 1: 9 ratio. After removing the excess coating liquid from the specimens, 1 ml of bacteria in nutrient broth was pipetted into each well. The well‐plates were then incubated under anaerobic conditions for and 24 h.

At 6 and 24 h, the test specimens were removed from the well plates and briefly dipped in 0.9% NaCl before the biofilm on the test surface was removed by vigorous swabbing with a cotton swab in a tube containing 0.5 ml of 0.9% NaCl. After mixing thoroughly by pipetting and exposing to ultrasound, a 10‐fold dilution series was made, and aliquots were plated on TSAB plates. The TSAB plates were incubated anaerobically for 10 days, after which the colony‐forming units (cfu) were counted. At 24 h, the biofilm “mass” and metabolic activity were also measured as previously described (Pirracchio et al. 2018).

### 2.5 Release of interleukin‐8 by Gingival Fibroblasts

Human gingival fibroblasts were donated by systemically healthy patients undergoing oral surgery. The patients were informed about the use of the tissue for research purposes and signed a written informed consent form. This procedure follows the guidelines of the Ethics Committee of the University of Bern; no additional approval from the Cantonal Ethics Committee (KEK) is needed for irreversibly anonymized biomaterial. The fibroblasts were handled according to the recently described procedure (Lin et al. 2020; Strauss et al. 2018). Fibroblasts between the third and fifth passages were used in the experiments. Fibroblasts from two donors were included.

Fibroblasts were transferred to 96‐well plates and grown to confluence in DMEM (Invitrogen, Carlsbad, CA, USA), supplemented with 10% fetal bovine serum (FBS; Invitrogen). A four‐fold dilution series of the coating solutions was prepared, starting from 50% of the ready‐to‐use solution. The solution was then mixed with DMEM at a ratio of 1:9 to achieve final concentrations of 5%, 1.25%, and 0.31%.

The bacterial suspension was prepared as described above and then exposed to ultrasonication. The disrupted bacteria are filtered through a 0.45 µm membrane to exclude viable bacteria.

The culture medium was removed from the fibroblasts. After two washes with phosphate‐buffered saline (PBS), DMEM containing diluted coating solutions and either 5% of bacterial filtrate or 0.9% w/v NaCl was pipetted into the wells. The plates containing the fibroblasts were incubated for 6 h with 5% CO_2_. The level of IL‐8 was determined using a commercially available ELISA kit (R&D Systems, Minneapolis, MN), following the manufacturer's instructions. The cutoff was 1 pg/ml.

Additionally, the viability of the gingival fibroblasts was analyzed using an MTT assay (Mosmann 1983) performed as previously described (Zhu et al. 2024).

### 2.6 Statistics

The primary outcome of the study was the difference in cfu counts after 24 h between the test and control groups. With an expected value of 7 ± 0.8 log10 cfu for the test/control group and a desired difference of 1 log10, seven samples per group were needed to achieve a power of 0.8.

In all biofilm and fibroblast experiments, eight independent values per group and variable were obtained in two series and entered into statistical analysis. All data were analyzed using SPSS 29.0 statistical software (IBM Corp., Armonk, NY, USA). ANOVA with post hoc Bonferroni was applied. Statistical significance was defined as *p* < 0.05.

## 3 Results

### 3.1 Minimum Inhibitory Concentration

The test solution clearly inhibited the growth of the selected oral bacteria. The greatest growth inhibition was observed with *A. naeslundii* ATCC 12104 at 0.1%. A concentration of 6.25% was necessary to block the multiplication of *S. mutans* ATCC 25175 and *C. rectus* ATCC 33238. The basic solution also acted as a growth inhibitor, but generally at higher concentrations. The minimum inhibitory concentrations of CHX were very low. All MIC values are shown in Table 1.

**Table 1:** Minimal inhibitory concentration (%) of the test and its basic solution as well as of the 0.2% chlorhexidine digluconate (CHX) solution against selected oral bacteria.

Strain	Test solution	Basic solution	CHX solution^a^
*Actinomyces naeslundii* ATCC 12104	0.10	2.50	0.10
*Streptococcus gordonii* ATCC 10558	3.13	25	0.10
*Streptococcus mutans* ATCC 25175	6.25	25	≤ 0.05
*Streptococcus sobrinus* ATCC 33478	3.13	12.5	≤ 0.05
*Fusobacterium nucleatum* ATCC 25586	3.13	25	≤ 0.05
*Parvimonas micra* ATCC 33270	3.13	25	0.20
*Campylobacter rectus* ATCC 33238	6.25	3.13	0.10
*Prevotella intermedia* ATCC 25611	3.13	3.13	0.10
*Tannerella forsythia* ATCC 43037	1.56	25	0.20

^a^ % are related to the 0.2% CHX solution.

### 3.2 Biofilm Results

**Figure 1:**
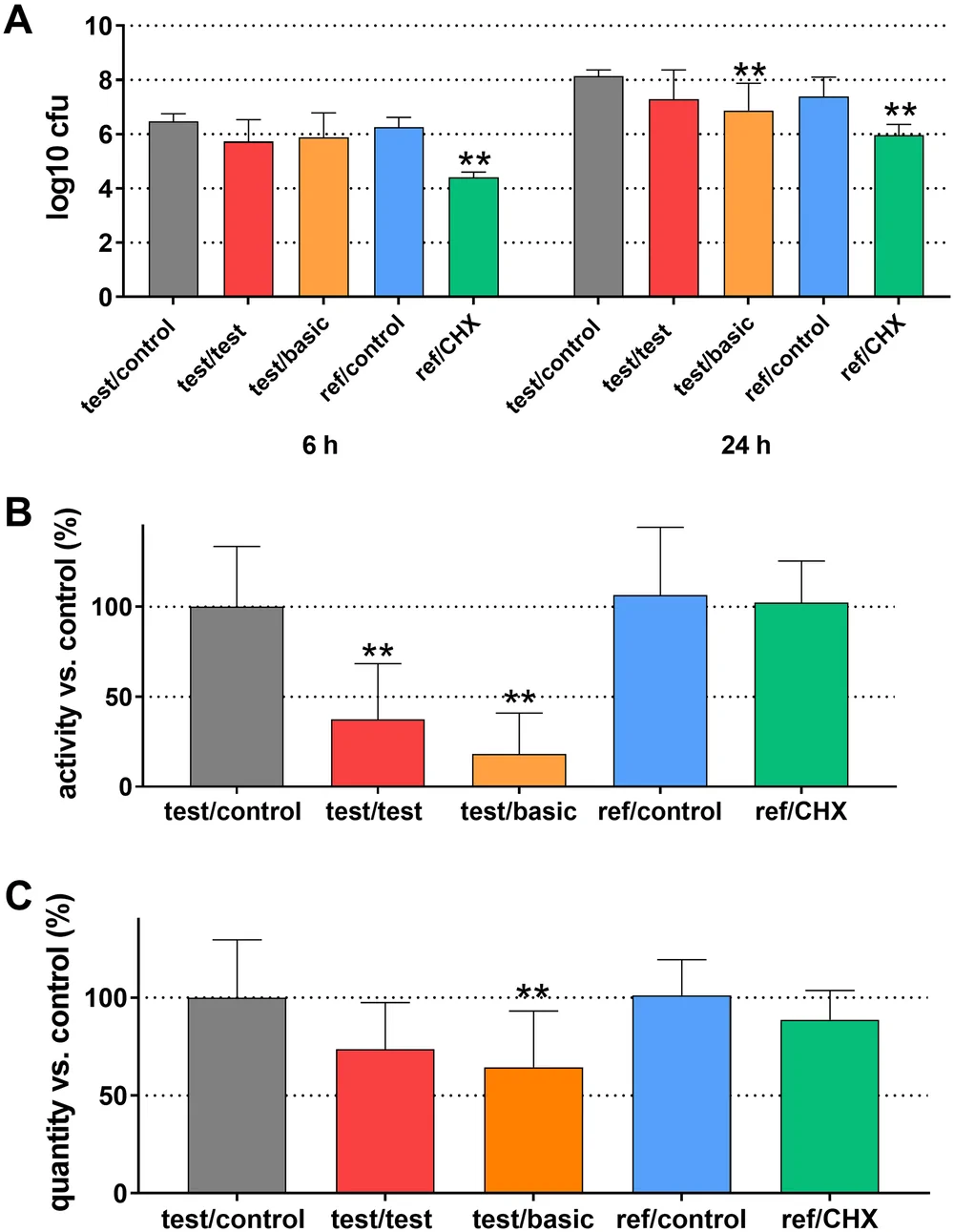
Colony forming units (cfu) after 6 h and 24 h of incubation (A), metabolic activity (B) and biofilm “mass” (C) after 24 h of incubation in the biofilms formed on the specimens consisting of test and reference (ref) material and coated with test, basic or 0.2% chlorhexidine digluconate (CHX) solution.

The results of the cfu counts after anaerobic cultivation were transformed to log10 and are shown in Figure 1A. After 6 h, the biofilm on the test material without a special coating contained in mean 6.41 ± 0.33 log10 cfu, the biofilm on the reference material 6.25 ± 0.37 log10 cfu. Coating of the reference material with CHX decreased the cfu counts by 0.83 log10 (*p* = 0.009 vs. the reference material). Counts on the test material decreased by 0.68 log10 cfu when coated with the test solution and by 0.53 log10 cfu with the basic solution; however, neither difference was statistically significant.

After 24 h, the biofilms on the test material without special coating contained in mean 8.14 ± 0.23 log10 cfu and the biofilms on the reference material contained in mean 7.39 ± 0.71 log10 cfu (not statistically significant). After 24 h, the cfu counts of attached bacteria decreased by 0.85 log10 cfu (not statistically significant) with the test solution and by 1.27 log10 cfu (*p* = 0.006) with the basic solution versus the test control. On the reference material, application of 0.2% CHX reduced the counts by 2.42 log10 cfu (*p* < 0.001). The difference between the reference material with the CHX solution and the test material with the test solution was significant (*p* = 0.003), but not between the test material with the basic solution and the reference material with the CHX solution.

Biofilm metabolic activity was determined by measuring the reduction of resazurin. The mean value of the reference material control was similar to that of the test material control (107.0% of the control). After applying the test solution, biofilm metabolic activity decreased to 37.4% (*p* = 0.001 vs. the control), and after applying the basic solution, it decreased further to 18.0% (*p* < 0.001 vs. the control). There was no decrease in metabolic activity after coating the reference material with 0.2% CHX compared to the reference material (Figure 1B).

The biofilm “mass” was measured after fixation and staining with crystal violet. The mean value of the reference material control was nearly equal to that of the control (101.1%). After applying the test solution, the biofilm mass was 73.7% (not statistically significant vs. the test control), and after the basic solution, it was 64.4% of the control (*p* = 0.005 vs. the test control). Coating the reference material with 0.2% CHX did not decrease the biofilm “mass” (Figure 1C).

Gingival fibroblasts were exposed to 0.31%, 1.25%, and 5% of the coating solutions for 6 h, partly in the presence of the bacterial mixture used for biofilm formation. Then, the supernatant was collected, and the level of IL‐8 was quantified.

**Figure 2:**
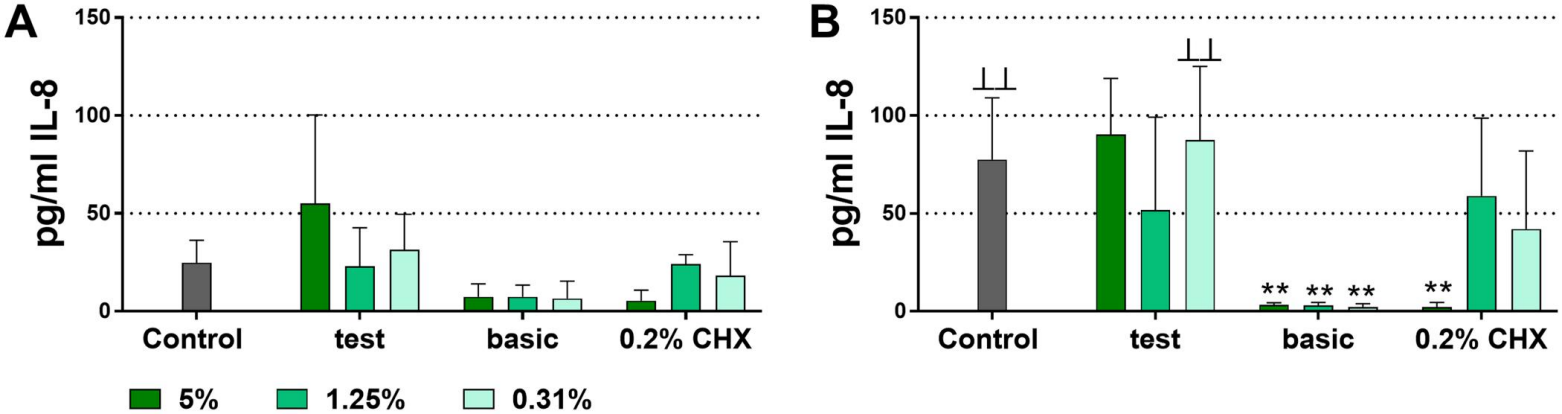
IL‐8 level in supernatants of gingival fibroblasts after 6 h incubation with 5%, 1.25% or 0.31% test, basic or 0.2% chlorhexidine digluconate (CHX) solutions without (A) and with (B) bacterial mixture. **p < 0.01 versus respective control. ^┴┴^ *p* < 0.01 versus respective group without bacterial exposure.

Without bacterial exposure, the added solutions did not statistically significantly change the release of IL‐8. In the control samples (without added coating solution), bacterial exposure increased IL‐8 levels (*p* = 0.002). Five percent of the 0.2% chlorhexidine (CHX) solution and all three tested concentrations of the basic solution decreased the level of IL‐8 in the presence of the bacterial mixture (each *p* < 0.001; Figure 2).

**Figure 3:**
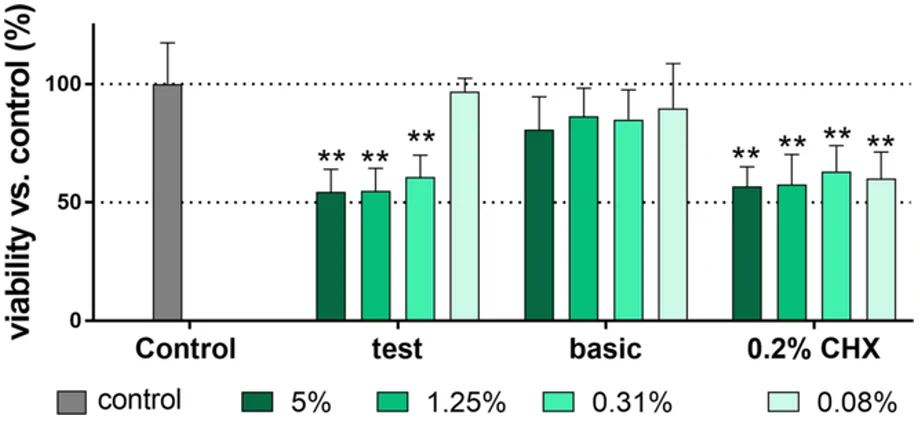
Cell viability of gingival fibroblasts after 60 min exposure of 0.08%, 0.31%, 1.25% and 5% of the test, basic or 0.2% chlorhexidine digluconate (CHX) solutions. **p < 0.01 versus control.

To determine the potential effect on cell viability, 0.08%, 0.31%, 1.25%, and 5% solutions were exposed to gingival fibroblasts. Cell viability decreased to 54.5%–62.6% after applying 0.08%–5% of the 0.2% CHX solution and 0.16%–5% of the test solution (each *p* < 0.001; Figure 3).

## 4 Discussion

In summary, the loading (test) solution showed a clear antimicrobial effect against oral bacteria. Applying the loading solution to a cellulose‐based thermoformable material reduced the metabolic activity of the formed biofilm. The solubilizing (basic) solution inhibited the growth of oral bacteria to a certain extent. Conversely, it exhibited strong anti‐biofilm activity. It reduced the release of the chemokine IL‐8 from gingival fibroblasts without affecting cell viability.

To specify a potential antibiofilm effect, we determined the direct antimicrobial activity of the solution against selected oral bacteria. Here we followed the standard recommendations for conventional testing of antibiotic resistance (Boattini et al. 2025). The loading solution suppressed the growth of the selected oral bacteria most effectively at low concentrations. This effect was more pronounced with the loading test solution than with the basic solution, suggesting a relationship with the supplemented EOs, such as cinnamaldehyde, eucalyptol, limonene, and trans‐anethole. These essential oils are widely used in medicinal and cosmetic formulations. Cinnamaldehyde, an active compound derived from cinnamon oil, is generally recognized as safe (Jaramillo Jimenez et al. 2024). Eucalyptol, which contains the active compound 1,8‐cineole, has been used in folk medicine for decades to treat respiratory diseases, for example (Cai et al. 2021). Limonene has demonstrated a broad spectrum of health benefits, including anxiolytic, anti‐inflammatory, and antioxidant activities, as well as analgesia (Lima et al. 2013; Vieira et al. 2018). It is often used as a flavoring agent and in perfume formulations (Ibáñez et al. 2020). Trans‐anethole, the main component of anise, has been used in traditional medicine for various therapeutic applications (Sharafan et al. 2022).

All of these EOs are well known for their antimicrobial activities (Jaramillo Jimenez et al. 2024). The antimicrobial properties are mainly due to their ability to cause cell membrane permeabilization (Hoch et al. 2023; Jaramillo Jimenez et al. 2024) At low concentrations, they suppress the growth of gram‐positive bacteria, such as *Staphylococcus aureus*, and gram‐negative bacteria, such as *Escherichia coli* and *Pseudomonas aeruginosa* (Nguyen et al. 2023; Soković et al. 2010; Yang et al. 2021). Regarding oral species, the MIC of cinnamaldehyde against *Actinomyces viscosus* was determined to be very low (Chen et al. 2024) which may explain our very low MIC value of the loading solution against *A. naeslundii*. An EO consisting of 90% limonene exhibited potent antibacterial activity against *S. mutans* (Torshabi et al. 2023).

The cellulose based test material was loaded with the EO solution and the basic solutions for 6 h. Recent research has found that optimal adsorption occurs within this time frame (Astasov‐Frauenhoffer et al. 2023). The results of the test solution on multi‐species biofilms showed a trend of fewer bacterial counts and reduced biofilm mass after 24 h, as well as clearly reduced metabolic activity. Other in vitro research has reported the inhibitory effects of limonene, eucalyptol, and trans‐anethole on *S. mutans* single‐species biofilm formation (Landeo‐Villanueva et al. 2023; Sun et al. 2018; Wongsariya et al. 2024). A few studies have shown a downregulation of gene expression; for example, limonene downregulates genes involved in initial adherence (Sun et al. 2018). Cinnamaldehyde decreased the biofilm mass and metabolic activity of both a *S. mutans* single‐species biofilm and a dual‐species biofilm combined with *Streptococcus sanguinis* (He et al. 2019). In principle, our results are consistent with those of other studies, albeit less notably. One possible explanation is the use of a multi‐species biofilm and the surface used for biofilm formation, as well as the mixture of the compounds. Coating the reference material with 0.2% chlorhexidine (CHX) yielded lower colony‐forming unit (CFU) counts related to the loading solution. However, the effect was relatively small, with a difference of only 2.4 log10, which is smaller than in recent research, where coating a polystyrene plate with a less concentrated CHX solution nearly completely blocked the formation of multispecies cariogenic, or periodontal biofilms (Stähli et al. 2021). This suggests that the reference material did not support the adsorption of the antimicrobial agent. CHX is well known for its ability to inhibit biofilm formation due to its high substantivity (Singh et al. 2022). There were no clear differences between the controls of the test and reference materials; only the CFU counts at 24 h tended to be higher for the test material, meaning that the test material did not negatively affect biofilm formation.

Unexpectedly, the basic solution used as a control (without supplemented essential oils) most effectively inhibited biofilm formation. An intensive search of the available literature revealed extremely limited data on the potential antimicrobial and antibiofilm effects of basic solution compounds. The basic solution contains polyglyceryl laurate/sebacate, a surfactant. In one study polyglyceryl laurate (PGL) did not negatively interfere with the antimicrobial activity of dissolved compounds (Park et al. 2020). Data are available on glycerol monolaurate (GML), a monoglyceride derivative. GML has a strong antimicrobial activity; like other medium chain fatty acids, its effect is based on membrane lysis ability and suppression membrane acids (Borrelli et al. 2021). GML exhibits strong inhibitory activity against gram‐positive bacteria (Kovanda et al. 2019). It inhibits the development of a three‐species biofilm containing *S. mutans* (Lester and Simmonds 2012) or affects the formation of *Staphylococcus epidermidis* biofilm without targeting bacterial viability (Krislee et al. 2019). In *Campylobacter jejuni* biofilms, lauric acid blocks the activity of auto‐inducer‐2 molecules being of importance in the interbacterial communication and biofilm formation (Li et al. 2021). These findings may support the results of the present study; however, specific data on the effects of polyglyceryl‐4 laurate on bacterial metabolism (beyond viability) and biofilm formation are lacking.

All of the added essential oils (EOs)—cinnamaldehyde, eucalyptol, limonene, and trans‐anethole—are used in therapeutics and cosmetics because of their anti‐inflammatory properties (Cai et al. 2021; Jaramillo Jimenez et al. 2024; Sharafan et al. 2022; Vieira et al. 2018). In the present study, we tested the effect of the solutions on the release of IL‐8, a chemokine that attracts neutrophiles (Matsushima et al. 2022), from gingival fibroblasts. We did not find an effect of the loading solution on IL‐8 release. However, the basic solution at all tested concentrations (0.31% ‐ 5%) and CHX at the highest concentration (5% of 0.2%CHX meaning 0.01% CHX) blocked IL‐8 release from gingival fibroblasts stimulated with biofilm. The release of IL‐8 from gingival fibroblasts induced by the four EOs has never been investigated. Cinnamaldehyde did not stimulate IL‐8 release in epithelial cells, lung fibroblasts (Gerloff et al. 2017) and an oral mucosa model (Hagi‐Pavli et al. 2014). Eucalyptol suppressed IL‐8 release from endothelial cells (Linghu et al. 2016). The results of the basic solution were unexpected. There is almost no published evidence on how PGL affects fibroblast (or general mammalian cell) metabolism (respiration, glycolysis, mitochondrial function) at non‐cytotoxic doses. GML inhibits IL‐8 synthesis induced by bacteria in human epithelial cells (Schlievert et al. 2019).

To rule out the possibility that the decrease in IL‐8 induced by the basic solution was a toxic effect on the gingival fibroblasts, the viability of the cells was determined. Since cell viability was not affected by the basic solution, the blocked IL‐8 release cannot be attributed to cytotoxicity. In contrast, all tested concentration of CHX decreased cell viability, which may explain the low IL‐8 level. Several assays have shown that CHX acts in a cytotoxic manner to a certain degree (Cunha et al. 2024; Dinu et al. 2024; Ivanova et al. 2023). Additionally, the loading solution affected the viability of the fibroblasts, and the effect was concentration‐dependent. Other research on essential oils (EOs) has reported different findings. For example, eucalyptol was highly cytotoxic to gingival fibroblasts (Puig‐Herreros et al. 2024). Dermal fibroblast viability decreased to less than 50% when exposed to 100 µg/ml trans‐cinnamaldehyde for 24 h (Ranjitkar et al. 2021), but limonene did not affect the dermal fibroblast viability (Kulig et al. 2022). The MTT test used in this study was applied to monolayers of gingival fibroblasts. A comparison of 2D monolayer cultures and 3D fibroblast spheroids revealed that the spheroids were 1.6 times more susceptible to cell death induced by nanoparticles than the monolayer cultures (Juarez‐Moreno et al. 2022). The cytotoxicity of sodium perborate‐containing formulations showed significant differences among monolayer cell cultures, 3D models, and an epidermis model (Leano et al. 2024). Therefore, translating the data into a clinical situation may be critical. Recently, the cytotoxicity of the loading solution was deemed acceptable according to European guidelines (Astasov‐Frauenhoffer et al. 2023).

This in vitro study has its advantages and limitations. Standard laboratory conditions were used, which enables comparison of the obtained results. The assays included not only the commercial product, but also the basic solution. A multispecies biofilm was formed. Bacterial filtrate, which is not viable bacteria, was in contact with gingival fibroblasts. Although the methods were adapted to the clinical situation, they only reflected a small part of the oral cavity's complexity. The biofilm consisted of nine defined bacterial strains and neglected individual differences. The oral microbiota is known to be diverse and to contain up to 700 species (Baker et al. 2024). Only monolayer cultures of gingival fibroblasts were used as host cells. Gingival fibroblasts are “nonclassical” members of the innate immune response (Wielento et al. 2023), “classical” immune cells such as macrophages, granulocytes, and T‐cells (Han et al. 2023), were not considered.

In conclusion, loading invisible dental braces with antimicrobial and anti‐inflammatory natural compounds, such as essential oils (EOs), may be an effective way to inhibit biofilm formation and suppress the development of caries and gingivitis. The PGL‐based solubilizer, in particular, exhibits anti‐biofilm and anti‐inflammatory properties. Further in vitro research is needed to better understand the solubilizer's mechanisms and optimize the composition of added EOs before testing in clinical trials.

## Author Contributions

S.E. and T.T. made substantial contributions to conception and design, M.L. to acquisition of data, A.St., A.Sc. A. St., A. Sc. and S.E. to analysis and interpretation of data. A.St., A. St., M.L., T.T. and S.E. were involved in drafting the manuscript. A.St., A.Sc. A. St., A. Sc. and S.E. revised it critically for important intellectual content. All authors gave final approval of the version to be published. Each author has participated sufficiently in the work to take public responsibility for appropriate portions of the content; and agrees to be accountable for all aspects of the work in ensuring that questions related to the accuracy or integrity of any part of the work are appropriately investigated and resolved.

## Conflicts of Interest

Tino Töpper is a former employee of Bottmedical AG. The other authors have no conflicts of interest to declare.

## Data Availability

The data that support the findings of this study are available from the corresponding author upon reasonable request. Data will be made available on request by the corresponding author.
